# Associations Among Depression, Self‐Compassion, and Learning Burnout in Nursing Students: A Three‐Wave Longitudinal Study

**DOI:** 10.1155/da/3758311

**Published:** 2026-03-05

**Authors:** Qinghua Wang, Xiaohong Cao, Tianjiao Du

**Affiliations:** ^1^ Institute of Foreign Languages, China Medical University, No. 77 Puhe Road, Shenyang North New Area, Shenyang, Liaoning Province, People′s Republic of China, cmu.edu.cn; ^2^ School of Marxism, China Medical University, No. 77 Puhe Road, Shenyang North New Area, Shenyang, Liaoning Province, People′s Republic of China, cmu.edu.cn; ^3^ School of Humanities and Social Sciences, China Medical University, No. 77 Puhe Road, Shenyang North New Area, Shenyang, Liaoning Province, People′s Republic of China, cmu.edu.cn

**Keywords:** depression, learning burnout, longitudinal study, nursing students, self-compassion

## Abstract

**Background:**

Previous cross‐sectional research shows that there were significant associations between depression, self‐compassion, and learning burnout. However, no longitudinal research has explored the temporal associations among depression, self‐compassion, and learning burnout in nursing students. The present study aimed to examine the temporal relationships among depression, self‐compassion, and learning burnout and to explore the longitudinal mediating role of self‐compassion between depression and learning burnout among nursing students.

**Methods:**

This is a three‐wave longitudinal study. Undergraduate nursing students (*N* = 494) from three medical universities in China participated in this study and completed online questionnaires three times in October of 2019, 2020, and 2021, respectively. Online questionnaires, including the Depression Anxiety Stress Scale‐21 (DASS‐21) depression subscale, the 26‐item Self‐Compassion Scale (SCS), the learning burnout scale (LBS), and demographic information. IBM SPSS (version 22.0) and Mplus (version 8.3) were used for data analyses.

**Results:**

Among nursing students, self‐compassion unidirectionally and negatively predicted depression, while learning burnout unidirectionally and positively predicted depression. The temporal relationship between self‐compassion and learning burnout was reciprocal and negative. Self‐compassion longitudinally mediated the relationship between learning burnout and depression.

**Conclusion:**

Learning burnout may lead to depression among nursing students. As a psychological resource, self‐compassion longitudinally negatively predicted both learning burnout and depression and could buffer the negative impact of learning burnout on depression. It is recommended that nursing educators identify and implement effective interventions to enhance nursing students’ self‐compassion to help them combat learning burnout and depression.

## 1. Introduction

Depression affects millions of people worldwide and is highlighted as the leading cause of disability by World Health Organization (WHO) [1]. Previous research shows that the prevalence of depression was high among nursing students [2–4]. Depression is a neurological condition characterized by low mood, inability to concentrate, loss of interest in activities, and feeling of extreme hopelessness [1]. Research shows that depression was negatively associated with self‐efficacy, self‐esteem, and life satisfaction while positively associated with anxiety, insomnia, and problematic media use among nursing students [4–7]. Nursing students with high depression levels were found to have lower academic performance, more tendency to drop out and higher risks of committing suicide [6, 8, 9]. As nursing students will become the nursing workforce in the future, there is an urgent need to identify the factors influencing depression among nursing students and implement effective interventions early.

Learning burnout is found to be another common mental health problem experienced by nursing students. Learning burnout refers to a kind of negative psychological state that students develop under long‐term stress and is manifested as dejection (feeling exhausted and losing enthusiasm for learning), improper behavior (feeling indifferent to and alienated from learning activities), and low achievement (feeling incompetent and a reduced sense of achievement in learning) [10]. Research shows that there was a high level of learning burnout among nursing students [11, 12]. Previous cross‐sectional research has already found the significantly positive relationship between learning burnout and depression among nursing students [13], but the temporal relationship between learning burnout and depression among nursing students has not been established yet. Therefore, the first aim of the present longitudinal study was to explore the temporal relationship between learning burnout and depression among nursing students.

Self‐compassion is a psychological resource in positive psychology that has caught researchers’ attention in the last two decades. First presented by Neff [14], the concept of self‐compassion refers to compassion towards oneself when facing difficult life circumstances or personal inadequacies. The construct consists of three main components: self‐kindness versus self‐judgment, common humanity versus isolation, and mindfulness versus over‐identification. In negative life situations, people with high self‐compassion levels treat themselves with care, warmth, and kindness rather than with harsh self‐judgment, place themselves in the context of humanity rather than in isolation, and adopt a balanced perspective toward sufferings rather than over‐identify with them [14]. Previous cross‐sectional research has already demonstrated the significantly negative association of self‐compassion with depression among nursing students [15, 16], but we did not find any research exploring the temporal relationship between self‐compassion and depression among nursing students. Thus, the second aim of the present longitudinal study was to explore the temporal relationship between self‐compassion and depression among nursing students. Moreover, although the significantly negative association of self‐compassion with learning burnout has also been demonstrated in previous cross‐sectional research [17], we did not find any research exploring the temporal relationship between self‐compassion and learning burnout among nursing students. Therefore, as the third aim of the present study, we tried to examine the temporal relationship between self‐compassion and learning burnout among nursing students by using a three‐wave longitudinal design.

According to the transactional model theory, stress arises in the dynamic interaction between the individual and the environment. In this interaction, psychological resources an individual possesses influence his assessment of the environment and the coping strategies he takes, which will in turn affect the individual’s perceived stress levels [18, 19]. As an important psychological resource, the protective role of self‐compassion against stress has already been demonstrated in previous research [20]. Self‐compassion has been found to play a mediating role between stress and learning burnout among high school students [21], and between stress and depression among higher education students [22]. However, to the best of our knowledge, no research has yet explored the relationships among depression, self‐compassion, learning burnout, and possible mediating role of self‐compassion between depression and learning burnout among nursing students. Since previous research has also demonstrated the effectiveness of self‐compassion and compassion‐based interventions in alleviating stress [23], depression [24], and burnout [25], we hypothesized that self‐compassion may have a longitudinal mediation effect on the relationship between depression and learning burnout among nursing students. Thus, the fourth aim of the present study was to examine the longitudinal mediating role of self‐compassion between depression and learning burnout among nursing students.

To sum up, the aims of the present three‐wave longitudinal study were to explore (1) the temporal relationship between learning burnout and depression; (2) the temporal relationship between self‐compassion and depression; (3) the temporal relationship between self‐compassion and learning burnout; and (4) the longitudinal mediating role of self‐compassion between depression and learning burnout among nursing students.

## 2. Methods

### 2.1. Study Design and Participants

Participants in this three‐wave longitudinal study were from three medical universities in China. We followed the group of undergraduate nursing students through 3 years from 2019 to 2021. Online questionnaires were distributed in October of the years 2019, 2020, and 2021, respectively, when nursing students were in their first, second, and third academic year of study. In October 2019, for Wave One, 558 first‐year nursing students (141 males and 417 females; *M*_age_ = 18.03 years, SD_age_ = 0.645 years, *T*_1_) agreed to participate and filled out the questionnaires. In October 2020, for Wave Two, 578 second‐year nursing students (155 males and 423 females; *M*_age_ = 19.22 years, SD_age_ = 0.621 years, *T*_2_) agreed to participate and filled out the questionnaires. In October 2021, for Wave Three, 561 third‐year nursing students (139 males and 422 females; *M*_age_ = 19.97 years, SD_age_ = 0.787 years, *T*_3_) agreed to participate and filled out the questionnaires. Among the participants, 494 nursing students (111 males and 383 females) completed questionnaires in all three waves and we used the data from this group of 494 nursing students for the cross‐lagged analyses in our study. For the homogeneity tests of 494 nursing students as the representative for 558, 578, and 561 participants, respectively, in the three waves, please refer to the Tables S1–S3.

### 2.2. Measures

#### 2.2.1. Depression

Nursing students’ depression was measured by the Depression Anxiety Stress Scale‐21 (DASS‐21) depression subscale [26]. DASS‐21 contains 21 items which fall into three subscales: depression (seven items, e.g., “I was unable to become enthusiastic about anything.”), anxiety (seven items, e.g., “I was aware of dryness of my mouth.”) and stress (seven items, e.g., “I tended to over‐react to situations.”). Participants rated each item on a four‐point Likert scale from 0 (did not apply to me at all) to 3 (applied to me very much or most of the time) based on the applicability of the mental state described by each item to their past week experience. For each subscale, the total score of the corresponding seven items was calculated with higher total scores indicating higher levels of depression, anxiety and stress. The Chinese version of DASS‐21 demonstrated sound psychometric properties in previous studies [27, 28]. In the present study, the internal consistency of the depression subscale as measured by Cronbach’s alpha coefficients were 0.774 at *T*_1_, 0.889 at *T*_2_, and 0.868 at *T*_3_, respectively.

#### 2.2.2. Self‐Compassion

The 26‐item self‐compassion scale (SCS) was used to measure nursing students’ self‐compassion levels. SCS was developed by Neff [14] and contains six subscales: self‐kindness (five items, e.g., “I’m kind to myself when I’m experiencing suffering.”) vs. self‐judgment (five items, e.g., “When times are really difficult, I tend to be tough on myself.”), common humanity (four items, e.g., “I try to see my failings as part of the human condition.”) vs. isolation (four items, e.g., “When I think about my inadequacies, it tends to make me feel more separate and cut off from the rest of the world.”), and mindfulness (four items, e.g., “When something upsets me, I try to keep my emotions in balance.”) vs. overidentification (four items, e.g., “When I’m feeling down, I tend to obsess and fixate on everything that’s wrong.”). The 13 items in the three subscales of self‐judgment, isolation and overidentification were negatively worded items and needed to be reversely scored when calculating the total score. On a 5‐point Likert scale, nursing students rated the frequency of the experience described by each item from 1 (almost never) to 5 (almost always). After the negatively worded items were reversely scored, the total score was calculated with higher total scores indicating higher levels of self‐compassion among nursing students. SCS has been widely used among Chinese university students and has demonstrated satisfactory reliability and validity [29, 30]. In the present study, the reliability of SCS as measured by Cronbach’s alpha coefficients were 0.887 at *T*_1_, 0.890 at *T*_2_, and 0.890 at *T*_3_, respectively.

#### 2.2.3. Learning Burnout

Nursing students’ learning burnout was measured by the learning burnout scale (LBS) [10]. LBS was developed and modified by Lian [10] based on Maslach’s burnout inventory [31]. LBS includes three subscales: dejection (eight items, e.g., “When I wake up in the morning, thinking about studying for the whole day, I feel exhausted.”), improper behavior (six items, e.g., “I rarely study by myself after class.”) and low achievement (six items, e.g., “I can catch up with my academic work in college.”). Among the 20 items, eight items were negatively worded and needed to be reversely scored when calculating the total score. Nursing students indicated the extent of conformity to the situation described by each item on a 5‐point Likert scale from 1 (not true of me at all) to 5 (very true of me). The total score was then calculated with a higher total score signifying a higher level of learning burnout. LBS has shown good psychometric properties among Chinese healthcare profession students in previous studies [32, 33]. In the present study, Cronbach’s alpha coefficients measuring the internal consistency of LBS were 0.863 at *T*_1_, 0.892 at *T*_2_, and 0.907 at *T*_3_, respectively.

### 2.3. Data Analyses

SPSS version 22.0 was employed to conduct descriptive statistics analysis, homogeneity tests, scale reliability analysis, Pearson correlation analysis, one way ANOVA, and post hoc tests. Mplus version 8.3 was used to construct a three‐wave cross‐lagged model to examine the temporal relationships among learning burnout, self‐compassion and depression, and the possible mediating effect of self‐compassion between learning burnout and depression among nursing students. For model fit, five indices were evaluated, including Chi‐square divided by degrees of freedom (*χ* ^2^/df), comparative fit index (CFI), Tucker Lewis index (TLI), root mean square error of approximation (RMSEA), and standardized root mean square residual (SRMR). When *χ* ^2^/df <5, CFI >0.90, TLI >0.90, RMSEA <0.08, and SRMR < 0.08, the model was of acceptable fit [34]. For mediation analysis, the bootstrap method with asymptotic and resampling strategies were used, and when 95% confidence interval for the indirect effect did not include zero, the indirect effect was considered significant [35]. All statistical tests were carried out with the significance level set at *p* < 0.05 (two‐tailed).

### 2.4. Ethical Considerations

The present study was approved by the ethics committee of the authors’ affiliated institution and was conducted in line with principles in the Declaration of Helsinki. Trained research investigators explained the purpose of the study before distributing online questionnaires. Nursing students were assured that participation was voluntary and they could withdraw from the study anytime without any punishment. Students scanned a QR code using their cellphones and filled out the informed consent forms and questionnaires online. The completion and submission of questionnaires indicated that nursing students agreed to participate in the study voluntarily.

## 3. Results

### 3.1. Correlations Between Depression, Self‐Compassion, and Learning Burnout at Three Time Points

Table 1 shows the means, standard deviations, and Pearson correlation coefficients of depression, self‐compassion, and learning burnout among nursing students at three measurement time points. As can be seen from the table, all three psychological variables were significantly correlated with each other (*p*  < 0.001) and in the expected directions. Specifically, depression was significantly negatively associated with self‐compassion (*r* range: −0.550 to −0.355) but was significantly positively associated with learning burnout (*r* range: 0.330–0.550) among nursing students. Self‐compassion was significantly negatively related to learning burnout (*r* range: −0.580 to −0.340) among nursing students.

**Table 1:** Means (*M*), standard deviations (SD), and correlations of depression, self‐compassion, and learning burnout at three time points.

Variables	1.	2.	3.	4.	5.	6.	7.	8.	9.
1. DE (*T*_1_)	1	—	—	—	—	—	—	—	—
2. DE (*T*_2_)	0.482 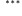	1	—	—	—	—	—	—	—
3. DE (*T*_3_)	0.497 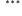	0.445 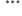	1	—	—	—	—	—	—
4. SC (*T*_1_)	−0.550 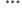	−0.449 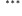	−0.411 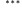	1	—	—	—	—	—
5. SC (*T*_2_)	−0.387 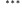	−0.526 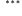	−0.390 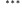	0.599 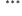	1	—	—	—	—
6. SC (*T*_3_)	−0.355 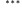	−0.366 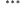	−0.514 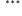	0.560 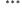	0.580 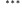	1	—	—	—
7. LB (*T*_1_)	0.550 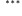	0.350 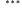	0.330 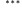	−0.495 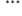	−0.418 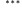	−0.340 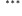	1	—	—
8. LB (*T*_2_)	0.442 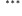	0.523 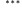	0.384 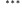	−0.434 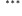	−0.576 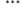	−0.436 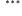	0.663 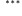	1	—
9. LB (*T*_3_)	0.362 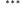	0.354 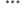	0.496 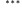	−0.427 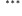	−0.468 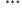	−0.580 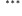	0.580 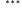	0.679 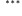	1
*M*	1.91	2.41	2.26	86.97	89.01	88.28	50.09	49.54	50.20
SD	2.40	3.15	2.97	12.54	12.45	11.44	9.83	11.15	10.85

*Note: N* = 494. *T*_1_, time point 1; *T*_2_, time point 2; *T*_3_, time point 3.

Abbreviations: DE, depression; LB, learning burnout; SC, self‐compassion.

^∗∗∗^ *p*  < 0.001 (two‐tailed).

### 3.2. Changes in Depression, Self‐Compassion, and Learning Burnout Over the Three Waves

Changes in depression, self‐compassion, and learning burnout among nursing students over the three waves were explored by using one way ANOVA and post hoc Bonferroni tests. The results, shown in Table 2, indicates that there were significant differences in levels of depression and self‐compassion among nursing students between *T*_1_ and *T*_2_. Specifically, nursing students’ depression and self‐compassion levels at *T*_2_ were both significantly higher than those at *T*_1_ (*p*  < 0.05), which means that there were significant increases in nursing students’ depression and self‐compassion levels as they changed from freshers to sophomores.

**Table 2:** Changes in depression, self‐compassion, and learning burnout over the three waves.

Variables	Time	Mean difference	*p*‐Value
Depression Self‐compassion Learning burnout	*T*_2_ − *T*_1_	0.500 2.047 −0.547	0.018 0.025 1.000
Depression Self‐compassion Learning burnout	*T*_3_ − *T*_2_	−0.154 −0.729 0.662	1.000 1.000 0.983
Depression Self‐compassion Learning burnout	*T*_3_ − *T*_1_	0.346 1.318 0.115	0.171 0.266 1.000

*Note: N* = 494. *T*_1_, time point 1; *T*_2_, time point 2; *T*_3_, time point 3.

### 3.3. Longitudinal Associations Among Depression, Self‐Compassion, and Learning Burnout

**Figure 1:**
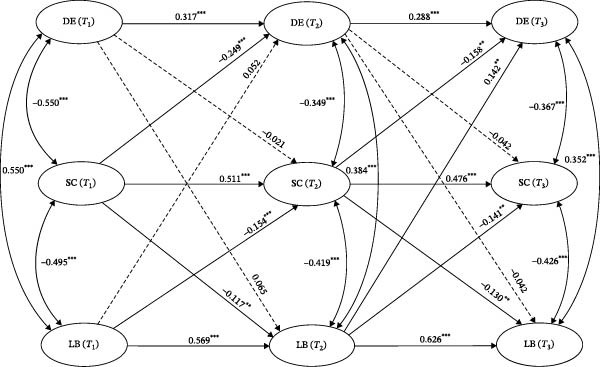
Three‐wave cross‐lagged model examining the longitudinal associations among depression, self‐compassion, and learning burnout in nursing students (*N* = 494). DE, depression; LB, learning burnout; SC, self‐compassion; *T*_1:_ time point 1; *T*_2:_ time point 2; *T*_3:_ time point 3. Path coefficients are standardized; 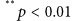 (two‐tailed); 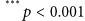 (two‐tailed). Dashed lines indicate nonsignificant relationships.

Figure 1 shows the three‐wave cross‐lagged model examining longitudinal associations among depression, self‐compassion, and learning burnout in nursing students. Results of the analysis indicate that the model was of acceptable fit, with *χ* ^2^ /df = 3.888, *p* = 0.0086, CFI = 0.995, TLI = 0.949, RMSEA = 0.077, and SRMR = 0.010. As can be seen from the Figure 1, the longitudinal relationship between depression and self‐compassion was unidirectional and negative. Specifically, self‐compassion at *T*_1_ negatively predicted depression at *T*_2_ (*β* = −0.249, *p*  < 0.001) and self‐compassion at *T*_2_ negatively predicted depression at *T*_3_ (*β* = −0.158, *p*  < 0.01). The longitudinal relationship between depression and learning burnout was also unidirectional but positive, with learning burnout at *T*_2_ significantly and positively predicting depression at *T*_3_ (*β* = 0.142, *p*  < 0.01). By contrast, the longitudinal relationship between self‐compassion and learning burnout was reciprocal and negative. To be specific, self‐compassion at *T*_1_ negatively predicted learning burnout at *T*_2_ (*β* = −0.117, *p*  < 0.01) and self‐compassion at *T*_2_ negatively predicted learning burnout at *T*_3_ (*β* = −0.130, *p*  < 0.01). Likewise, learning burnout at *T*_1_ also negatively predicted self‐compassion at *T*_2_ (*β* = −0.154, *p*  < 0.001) and learning burnout at *T*_2_ also negatively predicted self‐compassion at *T*_3_ (*β* = −0.141, *p*  < 0.01).

### 3.4. Longitudinal Mediating Role of Self‐Compassion Between Learning Burnout and Depression

The longitudinal mediating role of self‐compassion between learning burnout and depression among nursing students was explored by using the bootstrap method and results were presented in Table 3. It is evident from the Table 3 that the longitudinal mediating effect of self‐compassion on the relationship between learning burnout and depression among nursing students was significant (indirect effect: 0.024, SE: 0.010, 95% CI: 0.007, 0.041, *p* = 0.020).

**Table 3:** Longitudinal mediation analysis of self‐compassion between learning burnout and depression.

Indirect path	Indirect effect (SE)	95% CI	*p*‐Value
LB (*T*_1_) → SC (*T*_2_) → DE (*T*_3_)	0.024 (0.010)	0.007, 0.041	0.020

*Note: N* = 494.*T*_1_, time point 1; *T*_2_, time point 2; *T*_3_, time point 3.

Abbreviations: CI, confidence interval; DE, depression; LB, learning burnout; SC, self‐compassion; SE, standard error.

## 4. Discussion

The present study explored the temporal associations among depression, self‐compassion, and learning burnout in nursing students by adopting a three‐wave longitudinal design. Results show that self‐compassion unidirectionally and negatively predicted depression, while learning burnout unidirectionally and positively predicted depression. The temporal relationship between self‐compassion and learning burnout was reciprocal and negative. Specifically, self‐compassion negatively predicted learning burnout and learning burnout also negatively predicted self‐compassion over the three waves. Self‐compassion played a significant longitudinal mediating role between learning burnout and depression. These findings have important implications for nursing education and provide insights into implementing effective interventions to help nursing students maintain their mental health.

### 4.1. The Longitudinal Association Between Depression and Self‐Compassion

The present study found that self‐compassion negatively predicted depression among nursing students but not vice versa. This finding is in line with the conclusion of the study by Pang et al. [36], which found that self‐compassion negatively predicted depression among a group of adolescent boys 6 months later. Another study by Galvin et al. [37] also revealed that self‐compassion unidirectionally and negatively predicted depression among a group of autistic adults. However, the study by Pang et al. [36] did not find that self‐compassion could negatively predict depression among adolescent girls 6 months later. In addition, the study by Galvin et al. [37] revealed that the longitudinal relationship between self‐compassion and depression was bidirectional in nonautistic adults instead of unidirectional in autistic adults. Another longitudinal study also revealed the bidirectional and negative relationship between self‐compassion and depressive symptoms among a group of midlife women [38]. These mixed results on the longitudinal association between self‐compassion and depression require that further research needs to be done to clarify the temporal relationship between self‐compassion and depression especially among different population groups.

### 4.2. The Longitudinal Association Between Depression and Learning Burnout

Results on the temporal relationship between depression and learning burnout in previous longitudinal studies were also mixed. The two‐wave study by Zhang et al. [39] and the two‐wave study by Salmela‐Aro et al. [40] both found that the longitudinal association between depression and learning burnout was positive and reciprocal among adolescents. The three‐wave study by Kan et al. [41] adopting cross‐lagged panel network analysis also found that depression was an influential positive predictor of learning burnout among adolescents. However, the two‐wave study by Song et al. [42] did not find a significant longitudinal association between learning burnout and depression among adolescents. In the present study, learning burnout was found to predict depression positively and unidirectionally among nursing students from time point 2 to time point 3, but the longitudinal association between learning burnout and depression among nursing students from time point 1 to time point 2 was found to be nonsignificant. Until now, most previous longitudinal studies exploring the temporal relationship between depression and learning burnout have been conducted among adolescents. Therefore, more research is required to clarify the temporal relationship between depression and learning burnout among nursing students.

### 4.3. The Longitudinal Association Between Self‐Compassion and Learning Burnout

In the present study, the temporal relationship between self‐compassion and learning burnout was found to be negative and reciprocal among nursing students. In other words, self‐compassion significantly and negatively predicted learning burnout and, conversely, learning burnout also significantly and negatively predicted self‐compassion across three waves. Specifically, higher self‐compassion at time point 1 predicted lower learning burnout at time point 2. Higher self‐compassion at time point 2 also predicted lower learning burnout at time point 3. On the other hand, higher learning burnout at time point 1 predicted lower self‐compassion at time point 2. Higher learning burnout at time point 2 also predicted lower self‐compassion at time point 3. In previous longitudinal studies, the protective role of self‐compassion against burnout has already been established [43–45]. However, to the best of our knowledge, no longitudinal study has yet examined the temporal relationship between self‐compassion and learning burnout. The finding of the present study—that the temporal relationship between self‐compassion and learning burnout was negative and reciprocal—confirms the protective role of self‐compassion against burnout reported in previous studies. At the same time, this finding also warned us of the significantly negative impacts of learning burnout on self‐compassion among nursing students.

### 4.4. The Longitudinal Mediating Role of Self‐Compassion Between Learning Burnout and Depression

Another new finding of the present study is that self‐compassion longitudinally mediated the relationship between learning burnout and depression among nursing students. Lower levels of learning burnout at time point 1 was associated with higher levels of self‐compassion at time point 2, which in turn associated with lower levels of depression at time point 3. That is to say, self‐compassion longitudinally buffered the negative impacts of learning burnout on depression among nursing students. This finding further confirmed the protective role of self‐compassion against mental health problems such as learning burnout and depression. Although previous interventional studies have demonstrated the effectiveness of self‐compassion promotion training in alleviating burnout and depression [46–51], no study has explored the longitudinal mediating role of self‐compassion in the relationship between learning burnout and depression. The finding of the present study that self‐compassion longitudinally mediated the relationship between learning burnout and depression among nursing students indicated that nursing educators may implement effective interventions to promote nursing students’ self‐compassion in order to help them mitigate the negative influence of learning burnout on depression.

### 4.5. Implications for Nursing Education

The findings of the present study have important implications for nursing education. First, learning burnout unidirectionally and positively predicted depression among nursing students. This finding indicated that measures of lowering nursing students’ learning burnout levels may be effective in lowering their depression levels as well. Second, the finding that self‐compassion unidirectionally and negatively predicted depression among nursing students means that we can also help nursing students lower their depression levels by increasing their self‐compassion levels. Third, the longitudinal relationship between self‐compassion and learning burnout among nursing students was found to be reciprocal and negative. This finding suggested that enhancing self‐compassion levels would be helpful in reducing learning burnout levels and reducing learning burnout levels would also be helpful in enhancing self‐compassion levels among nursing students. Fourth, self‐compassion longitudinally mediated the relationship between learning burnout and depression, which indicated that improving self‐compassion helped buffer the negative impact of learning burnout on depression among nursing students. Based on these findings of the present study, it is recommended that nursing educators identify effective measures to raise nursing students’ self‐compassion levels in order to help them combat depression and learning burnout problems. Previous studies have demonstrated the efficacy of interventions, such as compassion focused therapy (CFT), cognitively‐based compassion training (CBCT), and compassion cultivation training (CCT) in enhancing participants’ self‐compassion levels and improving their mental health [52–54]. In addition, interventions, such as mindful self‐compassion (MSC), mindfulness‐based stress reduction (MBSR), and mindfulness‐based cognitive therapy (MBCT) have been demonstrated to be effective in reducing university students’ mental health problems through enhancing their mindfulness and self‐compassion levels [23, 55–58]. Therefore, nursing educators may consider implementing these interventions in nursing curricula to help raise nursing students’ self‐compassion levels and lower their learning burnout and depression levels.

### 4.6. Limitations and Future Directions

The present study has several limitations. First, the sample of nursing students in the present study came from only three medical universities in China, so generalizations about conclusions need to be made with caution. It is recommended that future multicenter studies in different cultures should be conducted. Second, we used self‐reported questionnaires as the data collection method, so response bias and social desirability bias cannot be avoided. Future studies may include qualitative data collection method to help reduce bias. Third, we only examined the associations of depression as measured by DASS‐21 depression subscale with self‐compassion and learning burnout. However, prior studies show that psychological distress, such as stress and anxiety, were also associated with self‐compassion and learning burnout, which can be examined in future studies. Fourth, when examining the associations among depression, self‐compassion, and learning burnout in nursing students, variables such as socioeconomic status, social support, coping skills, or specific academic variables were not measured. Future studies may explore the possible influence of these variables on depression, self‐compassion, and learning burnout. Fifth, although the present three‐wave longitudinal study explored the temporal associations and longitudinal mediation relationships among depression, self‐compassion, and learning burnout, conclusions on causal directions among these variables should be drawn with caution. It is recommended that future experimental studies with diagnostic and behavioral data be conducted to establish and further confirm causal relationships among the variables.

## 5. Conclusion

The present three‐wave longitudinal study explored the temporal associations among depression, self‐compassion, and learning burnout in nursing students. Results show that self‐compassion unidirectionally and negatively predicted depression, while learning burnout unidirectionally and positively predicted depression. As for the temporal association of self‐compassion with learning burnout, a reciprocal and negative relationship was found. Self‐compassion played a longitudinal mediating role between learning burnout and depression among nursing students. It is recommended that nursing educators identify and take effective measures to raise nursing students’ self‐compassion levels in order to help them combat depression and learning burnout problems.

## Author Contributions

Qinghua Wang was responsible for the study design, questionnaire survey, drafting, and revising the manuscript. Xiaohong Cao helped with data collection and Tianjiao Du provided some valuable advice.

## Funding

No funding was received for this manuscript.

## Disclosure

All the authors have read and approved the final version of the manuscript.

## Ethics Statement

The present study was approved by the Ethics Committee of China Medical University (Number 2019079) and was conducted in line with principles in the Declaration of Helsinki.

## Conflicts of Interest

The authors declare no conflicts of interest.

## Supporting Information

Additional supporting information can be found online in the Supporting Information section.

## Supporting information


**Supporting Information** Table S1. It shows results of homogeneity tests for Wave One. Table S2. It shows results of homogeneity tests for Wave Two. Table S3. It shows results of homogeneity tests for Wave Three.

## Data Availability

The data that support the findings of this study are available from the corresponding author upon reasonable request.
