# Supplementary material for: Associations Among Depression, Self‐Compassion, and Learning Burnout in Nursing Students: A Three‐Wave Longitudinal Study
Source: Depress Anxiety. 2026 Mar 5;2026:3758311. doi: 10.1155/da/3758311 (PMC12963458; doi:10.1155/da/3758311)
Supplement: Supplementary file 1 — Supporting Information Table S1. It shows results of homogeneity tests for Wave One. Table S2. It shows results of homogeneity tests for Wave Two. Table S3. It shows results of homogeneity tests for Wave Three. [file DA-2026-3758311-s001.doc]

**Supplementary Table 1: Results of homogeneity tests for Wave One**

| Variables | Group 1 (*N* = 558)  (M ± SD)/(n, %) | Group 2 (*N* = 494)  (M ± SD)/(n, %) | t/χ2 | *P* |
| --- | --- | --- | --- | --- |
| Age | 18.03 ± 0.645 | 18.04 ± 0.639 | -0.388 | 0.698 |
| Gender |  |  | 1.127 | 0.288 |
| Male | 141 (25.3%) | 111 (22.5%) |  |  |
| Female | 417 (74.7%) | 383 (77.5%) |  |  |
| Learning burnout | 50.05 ± 9.924 | 50.09 ± 9.830 | -0.063 | 0.950 |
| Self-compassion | 86.64 ± 12.659 | 86.97 ± 12.540 | -0.421 | 0.674 |
| Depression | 1.99 ± 2.534 | 1.91 ± 2.398 | 0.513 | 0.608 |

Note. M ± SD: mean ± standard deviation; t: independent samples t test ; χ2: Pearson chi-squared test.

**Supplementary Table 2: Results of homogeneity tests for Wave Two**

| Variables | Group 1 (*N* = 578)  (M ± SD)/(n, %) | Group 2 (*N* = 494)  (M ± SD)/(n, %) | t/χ2 | *P* |
| --- | --- | --- | --- | --- |
| Age | 19.22 ± 0.621 | 19.22 ± 0.613 | -0.124 | 0.902 |
| Gender |  |  | 2.698 | 0.100 |
| Male | 155 (26.8%) | 111 (22.5%) |  |  |
| Female | 423 (73.2%) | 383 (77.5%) |  |  |
| Learning burnout | 49.77 ± 11.176 | 49.54 ± 11.145 | 0.333 | 0.739 |
| Self-compassion | 88.55 ± 12.412 | 89.01 ± 12.452 | -0.602 | 0.547 |
| Depression | 2.62 ± 3.455 | 2.41 ± 3.145 | 1.043 | 0.297 |

Note. M ± SD: mean ± standard deviation; t: independent samples t test ; χ2: Pearson chi-squared test.

**Supplementary Table 3: Results of homogeneity tests for Wave Three**

| Variables | Group 1 (*N* = 561)  (M ± SD)/(n, %) | Group 2 (*N* = 494)  (M ± SD)/(n, %) | t/χ2 | *P* |
| --- | --- | --- | --- | --- |
| Age | 19.97 ± 0.787 | 19.97 ± 0.786 | 0.085 | 0.932 |
| Gender |  |  | 0.774 | 0.379 |
| Male | 139 (24.8%) | 111 (22.5%) |  |  |
| Female | 422 (75.2%) | 383 (77.5%) |  |  |
| Learning burnout | 50.39 ± 10.934 | 50.20 ± 10.849 | 0.280 | 0.780 |
| Self-compassion | 87.83 ± 11.681 | 88.28 ± 11.440 | -0.642 | 0.521 |
| Depression | 2.40 ± 3.118 | 2.26 ± 2.966 | 0.785 | 0.433 |

Note. M ± SD: mean ± standard deviation; t: independent samples t test ; χ2: Pearson chi-squared test.
